# ResMarkerDB: a database of biomarkers of response to antibody therapy in breast and colorectal cancer

**DOI:** 10.1093/database/baz060

**Published:** 2019-06-04

**Authors:** Judith Pérez-Granado, Janet Piñero, Laura I Furlong

**Affiliations:** Research Programme on Biomedical Informatics (GRIB), IMIM (Hospital del Mar Medical Research Institute), UPF (Pompeu Fabra University), Dr. Aiguader, Barcelona, Spain

## Abstract

The clinical efficacy of therapeutic monoclonal antibodies for breast and colorectal cancer has greatly contributed to the improvement of patients’ outcomes by individualizing their treatments according to their genomic background. However, primary or acquired resistance to treatment reduces its efficacy. In this context, the identification of biomarkers predictive of drug response would support research and development of new alternative treatments. Biomarkers play a major role in the genomic revolution, supporting disease diagnosis and treatment decision-making. Currently, several molecular biomarkers of treatment response for breast and colorectal cancer have been described. However, information on these biomarkers is scattered across several resources, and needs to be identified, collected and properly integrated to be fully exploited to inform monitoring of drug response in patients. Therefore, there is a need of resources that offer biomarker data in a harmonized manner to the user to support the identification of actionable biomarkers of response to treatment in cancer. ResMarkerDB was developed as a comprehensive resource of biomarkers of drug response in colorectal and breast cancer. It integrates data of biomarkers of drug response from existing repositories, and new data extracted and curated from the literature (referred as ResCur). ResMarkerDB currently features 266 biomarkers of diverse nature. Twenty-five percent of these biomarkers are exclusive of ResMarkerDB. Furthermore, ResMarkerDB is one of the few resources offering non-coding DNA data in response to drug treatment. The database contains more than 500 biomarker-drug-tumour associations, covering more than 100 genes. ResMarkerDB provides a web interface to facilitate the exploration of the current knowledge of biomarkers of response in breast and colorectal cancer. It aims to enhance translational research efforts in identifying actionable biomarkers of drug response in cancer.

## Introduction

The heterogeneity of cancer at different levels, namely genetic, proteomic, morphological and even at the tumour microenvironment, poses challenges to its diagnosis and treatment (1). The development of therapeutic monoclonal antibodies (mAbs) for cancer treatment has improved patients’ outcomes by tailoring their treatments according to their genomic background (2). Currently, there are seven Food and Drug Administration (FDA)-approved mAbs for the treatment of breast and colorectal cancer, which are among the most commonly occurring cancer in women and men, respectively (3). While all the mAbs used for breast cancer treatment (trastuzumab, pertuzumab and trastuzumab emtansine) target HER2, the mAbs currently used for colorectal cancer treatment target Epidermal Growth Factor Receptor (EGFR) (cetuximab, panitumumab) or Vascular Endothelial Growth Factor (VEGF) (bevacizumab and ramucirumab). Nonetheless, primary or acquired resistance is frequently observed for targeted therapies (4, 5). So far, the molecular mechanisms of resistance to anti-HER2 mAbs have not been identified yet. Thus, candidate patients are selected according to amplification or over-expression of HER2. Regarding colorectal cancer, the anti-EGFR antibodies cetuximab and panitumumab are used to treat RAS wild-type colorectal cancer, but their efficacy is limited due to the emergence of acquired drug resistance. Therefore, the availability of prognostic biomarkers of treatment response would promote a better management of patients by means of more tailored treatments according to their needs (6).

Although several databases contain information on genomic alterations in cancer, there is a lack of resources exclusively focused on biomarkers of treatment response. Moreover, the data on biomarkers is not always structured, differs in the granularity of the information provided and is annotated with different terminologies. All these issues hinder the identification and prioritization of biomarkers to improve treatment of patients. To address these challenges, we have developed ResMarkerDB as a centralized repository that harmonizes data of biomarkers of response to FDA-approved mAbs for breast and colorectal cancer. To this end, we have integrated data from four publicly available repositories with information extracted from the literature by text mining followed by expert curation. Biomarker information in ResMarkerDB can be browsed according to the level of evidence supporting it (e.g. preclinical versus clinical studies) to aid in the prioritization of biomarkers of response to therapeutic mAbs. In addition, all the information is provided with their provenance (e.g. original source of the data). ResMarkerDB aims to promote the identification of existing and new actionable biomarkers of drug response in breast and colorectal cancer by making this knowledge accessible to both basic researchers and clinical practitioners. This resource is publicly available at http://www.resmarkerdb.org under the Creative Commons 4.0 license.

## Implementation

### Data collection

We extracted information on biomarkers of treatment response from the following resources: Cancer Genome Interpreter or CGI (v.2018/07/16) (7), Clinical Interpretations of Variants in Cancer or CIViC (v.2018/07/16) (8), JAX-Clinical Knowledgebase or JAX-CKB (v.2018/07/03) (9) and non-coding RNAs (ncRNAs) in Drug Resistance or ncDR (v.2016/06/28) (10) (Supplementary Table S1). Additionally, a new data set, ‘ResCur’, that contains expert-curated data extracted from the literature and ncDR by text mining was developed. The text mining information was extracted from PubMed abstracts using the tools Pubtator (11) and SCAIView (12). We focused on ncRNAs and point mutations. Publication retrieval and recognition of drug names, microRNA (miRNA), level of evidence and response were performed with SCAIView, while additional entities (tumour types, mutations, species and genes) were annotated using Pubtator. Finally, the text mining results were expert-curated by checking if all the entities were properly annotated, adding additional information and reviewing if the supporting statements were properly selected. Moreover, ncDR data were further annotated using Pubtator followed by expert curation to specify the ncRNA alteration and the specific cancer subtype, information not originally provided by ncDR.

### Data homogenization and standardization

**Figure 1:**
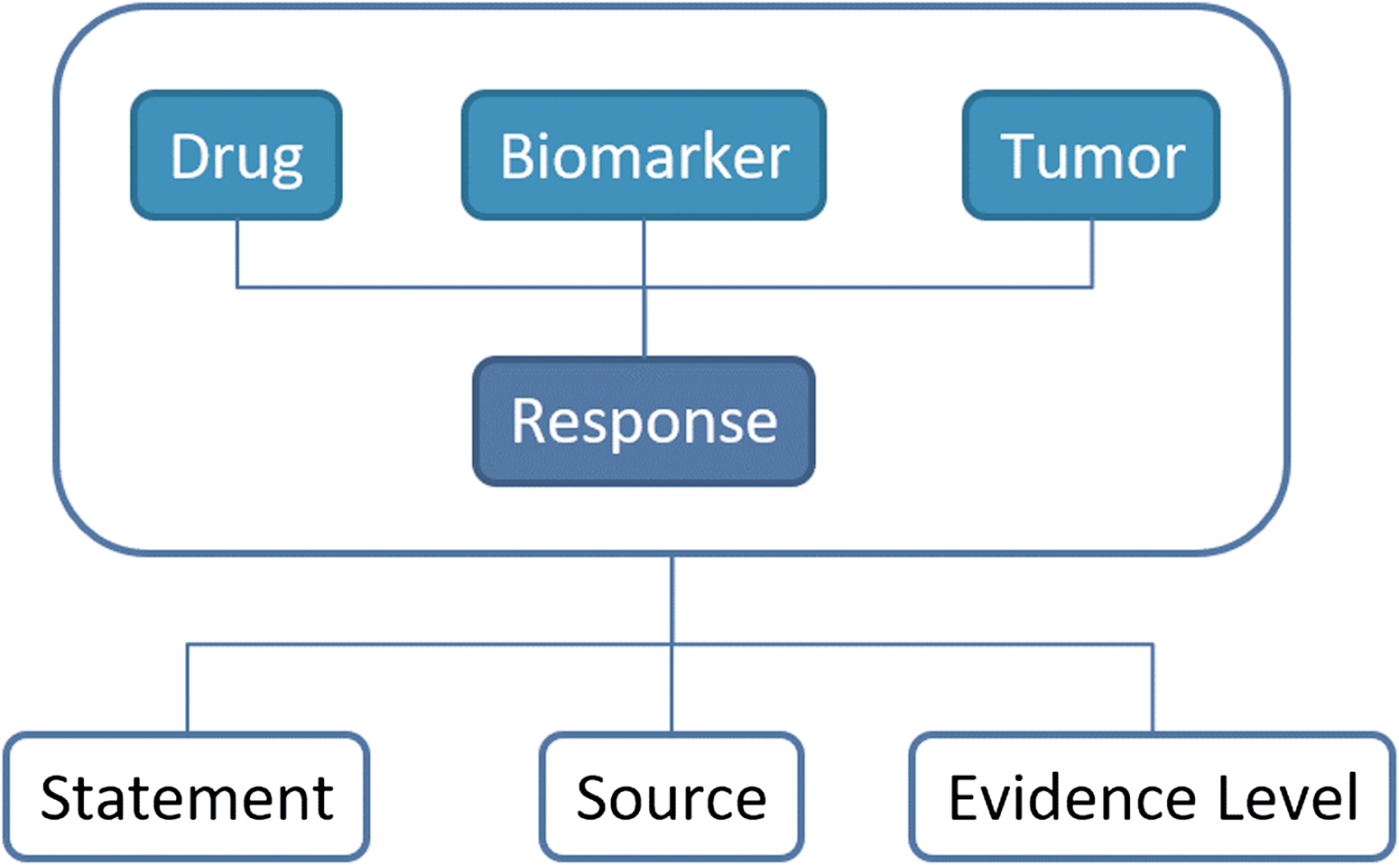
Schema of the database contents and their relationships. The information in ResMarkerDB is organized around the combination of biomarker and the response to a certain treatment in a certain cancer type. For this combination of entities, a supporting statement, the reporting source and the evidence level is provided.

After data collection, we conducted a process of data homogenization and standardization using ontologies and controlled vocabularies. This process was performed for each entity annotated in ResMarkerDB: biomarker, drug, tumour type, response, evidence level, source and statement. The information available in ResMarkerDB is centred in a biomarker and the associated response to a given treatment in a certain cancer type. For this combination (biomarker-drug-tumour-response), the level of supporting evidence, the original reporting source and a supporting statement from a publication are reported (Figure 1).

The original databases include biomarkers of diverse nature, including genomic elements such as mutations, copy number alterations (CNAs) and ncRNAs, but also processes such as changes in gene expression or in the localization of intracellular proteins. For this reason, we created the following categories to classify biomarkers in ResMarkerDB (Table 1): (i) gene variants (GVs), including alterations in the gene sequence, such as mutations and small insertions and deletions; (ii) CNAs, including gene amplifications and deletions; (iii) ncRNAs, such as miRNAs; (iv) expression alterations (EAs), such as changes in gene or protein expression, namely overexpression and underexpression; and (v) functional events (FEs), including nuclear translocation of proteins. Human single nucleotide variants were referenced to their corresponding Single Nucleotide Polymorphism database (dbSNP) identifiers using Biomart Ensembl tool, Ensembl Genes and Variation version 91 (13); while genes were referred with NCBI Entrez Gene identifiers (14).

**Table 1:** Classification of biomarkers according to their nature: protein coding genes (GVs, alone or in combination with other types of alterations: EAs, CNAs, FEs) and non-coding DNA (miRNA and lncRNA).

Protein coding genes	Gene variants	GV	126
		GV + GV	24
		GV + CNA	20
		GV + EA	7
		GV + EA + CNA	1
			178
	Other types	EA	18
		CNA + EA	6
		CNA + CNA	6
		CNA	6
		EA+ EA	5
		FE	1
			42
Non-coding DNA	miRNA	EA	45
	lncRNA	EA	1
			46
Total			266

ResMarkerDB focuses on FDA-approved mAbs for breast cancer (trastuzumab, pertuzumab and trastuzumab emtansine) and colorectal cancer (cetuximab, panitumumab, bevacizumab and ramucirumab) and also includes other agents such as antineoplastic, immunomodulating drugs and other mAbs that are usually administered in combination with the target mAbs. Drugs were cross-referenced with DrugBank identifiers (15). Moreover, they were classified according to the group ‘L antineoplastic and immunomodulating agents’ from the Anatomical Therapeutic Chemical (ATC) classification system (16). When the chemotherapeutic drug was not specified by the source database, we manually annotated it from the original reference. ResMarkerDB reports a total of 134 treatments and 73 drugs. Only three of those drugs were shared among all sources: trastuzumab, panitumumab and cetuximab (Supplementary Figure S1). The classification of the drugs according to the ATC system is shown in Supplementary Table S2, indicating that almost all drugs belong to the group of ‘L01 antineoplastic agents—L01X other antineoplastic agents’, being the majority ‘L01XE protein kinase inhibitors’ and ‘L01XC monoclonal antibodies’. We also have evidence of four drugs belonging to ‘L02 endocrine therapies’ and one to ‘L04 Immunosuppressant’.

**Figure 2:**
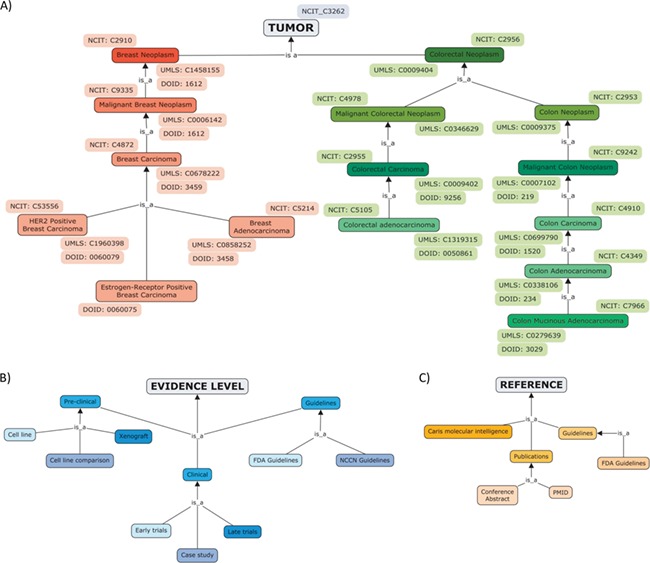
Hierarchical organization of contents of ResMarkerDB: (**A**) tumour types (breast and colorectal cancer), (**B**) levels of evidence (preclinical, clinical or guidelines mainly) and (**C**) sources.

The diseases were annotated with the NCI Thesaurus OBO Edition (NCIT), an ontology that covers cancer-related terminology including diseases, findings and abnormalities (17) (Figure 2A). NCIT provides a reference name, a description, a set of synonyms and a hierarchy of terms. In addition to the NCIT identifier, cross-references to Unified Medical Language System (18) and to Disease Ontology (19) were added to tumour types.

The database also includes the treatment response (sensitive or resistant) and evidence level, including preclinical studies (experiments in cell lines and xenograft), clinical studies, e.g. early trials (i.e. safety and toxicity studies) and late trials (i.e. efficacy studies) and guidelines (Figure 2B).

The reporting source refers to the original database where data was collected from and the evidence supporting the association. The latter includes publications and guidelines and tumour profiling companies (i.e. Caris Molecular Intelligence) (Figure 2C). If reported, a direct access to the publication in NCBI Pubmed, to the conference abstract in the Journal of Clinical Oncology or to the clinical trial was provided.

Finally, for each combination biomarker-drug-tumour-response we identified a sentence in the original publication that supported the association (the ‘supporting statement’). These statements were identified by text mining and manually selected when they were not provided by the source databases. In this way, the user can easily inspect the evidence from the original publication that reports the association, to have a direct access to the original evidence reported in the study (e.g. characterization of the tumour, specific drug regimen administration, etc.).

### Database creation and web page development

We have used the graphical database tool and database designer and creator MySQL Workbench (v6.3) and MariaDB as a database server (v5.5).

Django (v.1.11) (20), a high-level Python Web Framework, was used to develop the webpage platform, in addition to HTML5. Moreover, JavaScript and jQuery were also used. The application runs in an Apache Server (v.2.4).

### Data analysis

A functional enrichment analysis was performed using Panther (21) and the Gene Ontology (GO), with Benjamini–Hochberg false discovery rate (FDR) multiple test correction. Only results with FDR < 0.05 were considered. In addition, the coverage of the biomarkers included in ResMarkerDB was assessed on breast and colorectal cancer cohorts of patients from the Cancer Genome Atlas (TCGA). To this end, we used data corresponding to cohorts of breast carcinoma (1097 samples) (22) and colorectal carcinoma (170 samples) (23) accessible via the ‘TCGAbiolinks’ and ‘maftools’ R packages (24, 25). Finally, the ncRNAs were analysed for their association with breast and colorectal cancer using DisGeNET version 5.0 (26).

## Results

ResMarkerDB was developed as a web-based platform to facilitate exploration of current knowledge of biomarkers of response to FDA-approved mAbs in breast and colorectal cancer. It allows exploration, visualization and prioritization of biomarkers in the context of response to therapy. The data are provided with their ‘evidence level’, the reporting ‘source’ and a ‘statement’ supporting the evidence that helps in the assessment of the potential clinical impact (see Figure 1 and section *Data homogeneization and standardization* for more details).

ResMarkerDB contains 266 biomarkers of diverse nature (GVs, CNAs, EAs, FEs and ncRNAs) as single alterations or combinations of them (Table 1). GV is the most abundant biomarker type, representing 67% (178/266) of the biomarkers reported, alone or in combination with other alterations. ncRNAs represent 17% of the biomarkers in ResMarkerDB, being the 98% alterations of expression of miRNAs. Regarding the data sources, half of the collected biomarkers were provided by ‘JAX-CKB’. Moreover, 25% of the biomarkers are specific of ResCur, and from those, almost 70% are alterations in the expression of ncRNAs. Overall, there is a small fraction of biomarkers shared by all the sources (Supplementary Figure S1). The sources sharing the highest fraction of biomarkers are ‘CIViC’ and ‘JAX-CKB’. There are only nine biomarkers shared by ‘CGI’, ‘CIViC’ and ‘JAX-CKB’, four of which are also captured by ‘ResCur’. The small overlap of biomarkers among all the resources (4/266) is a consequence of differences in focus and annotation in the source databases. This highlights the need of integration of the information to offer a centralized repository specifically devoted to store biomarkers of treatment response.

A functional enrichment analysis was performed to characterize the genes encoding the ResMarkerDB biomarkers. This gene set is characterized by the biological processes *angiogenesis* (Fold Enrichment or F.E. = 40.31, *P*-value = 1.37E-03 and FDR = 1.45E-02) that is associated to metastasis, *I-kappaB kinase/NF-kappaB cascade* (F.E. = 24.19, *P*-value = 3.12E-04 and FDR = 3.81E-03), involved in inflammation and cell survival; *negative regulation of apoptotic processes* (F.E. = 21.99, *P*-value = 4.31-07 and FDR = 1.32E-05); and *MAPK cascade* (F.E. = 13.87, *P*-value = 1.03E-11 and FDR = 1.26E-09) involved in mitogenic signalling, among others (Supplementary Table S3). This analysis reveals a role of inflammation cascades, which enhance the acquisition of hallmark capabilities (27); alterations in cell division; as well as a negative regulation of apoptosis. Other enriched biological process were *transmembrane receptor protein tyrosine kinase signalling pathway*, related to regulation of cell cycle and cell growth, promoting tumour progression and often altering cell survival and energy metabolism; and *cell differentiation*, an important aspect for gene expression profile definition and tumour aggressiveness characterization (27).

**Figure 3:**
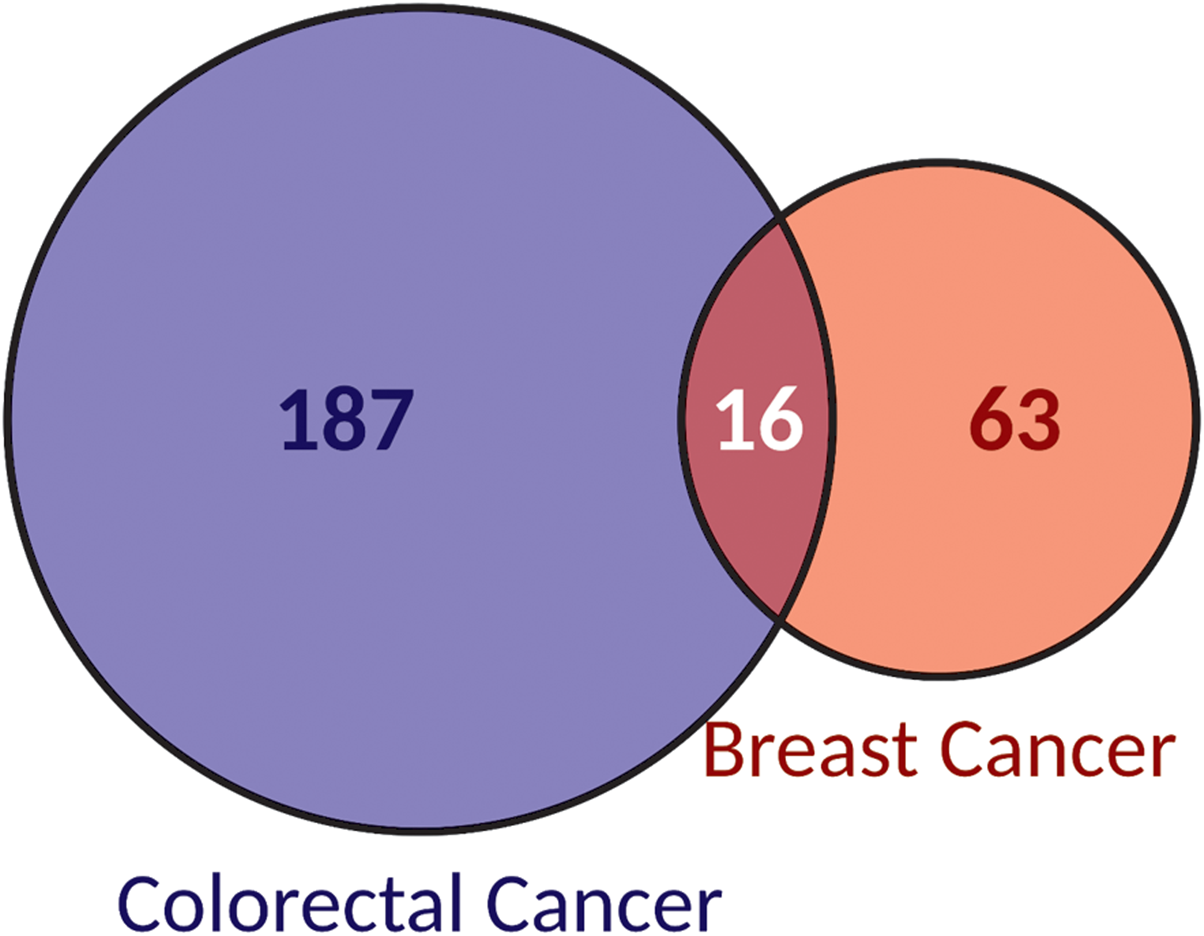
Classification of biomarkers according to the cancer type they are reported as associated to: breast or colorectal cancer.

Seventy percent of biomarkers (187/266) were specific to colorectal cancer while 24% (63/266) were specific to breast cancer. The remaining 6% of the biomarkers were associated to both cancer types (Figure 3). ERBB2, the target of the main mAbs used to treat breast cancer, is the gene most frequently reported as a biomarker of response in breast cancer, accounting for 59% of biomarkers with 23 different variants associated. It is followed by PIK3CA, with 20% of biomarker alterations. Although, PIK3CA is not found in treatment guidelines for breast cancer, as is the case for ERBB2, ESR1 or PGR, it is an oncogene found downstream ERBB2. Moreover, it has been characterized as a cancer driver gene in breast cancer (28) and is frequently reported as altered in this cancer type.

Alterations in both, ERBB2 and PIK3CA, cover 14% of biomarkers associated to breast cancer. Regarding colorectal cancer, KRAS is involved in 24% of the biomarkers of the database, spanning 29 different variants. It is followed by alterations in BRAF, PIK3CA and EGFR, considered in 15%, 12% and 10% of biomarkers, respectively. Contrarily to ERBB2, KRAS is not a target of the mAbs used in colorectal cancer. Panitumumab and cetuximab are two of the most commonly used mAbs in colorectal cancer and they target EGFR. Mutations in effectors downstream EGFR signalling pathway, especially in KRAS, have been described as negative predictors for anti-EGFR therapy (29).

An assessment of the fraction of patients with breast and colorectal cancer that harbour ResMarkerDB biomarkers was performed using data from the TCGA project. Thirty-six percent of the patients in a breast carcinoma cohort (395/1097 patients) have alterations in ResMarkerDB genes. In particular, 73% of these patients have mutations in the PIK3CA gene (Supplementary Figure S2). Furthermore, 69% (25/36) of ResMarkerDB biomarker genes were altered in this TCGA cohort. In the colorectal carcinoma TCGA cohort, 74% of the patients (126/170 patients) bear mutations in ResMarkerDB genes. Seventy-three percent of those patients have mutations in TP53, followed by 40% of the patients with mutations in KRAS, 16% in PIK3CA, 4% in BRAF and 2% in EGFR (Supplementary Figure S2). KRAS, BRAF and PIK3CA act downstream EGFR pathway and are therefore expected to be altered in drug response phenotypes (29).

The analysis of the biomarker-tumour associations per source shows that the highest number of associations shared by two different databases is found between ‘CIViC’ and ‘JAX-CKB’ (23 associations) (Supplementary Figure S3). Only four biomarker-tumour associations are shared between ‘CIViC’, ‘CGI’, ‘JAX-CKB’ and ‘ResCur’, being all of them associated to colorectal cancer (Supplementary Figure S3).

In the case of ncRNAs-tumour associations, 22% percent were related to breast cancer and 89% to colorectal cancer. The analysis of those ncRNAs in the context of disease, showed that 90% of breast cancer ncRNAs in ResMarkerDB are associated to different breast cancer types (Supplementary Figure S4), and 49% of ncRNAs biomarkers for colorectal cancer are associated to different colorectal cancer types according to DisGeNET (Supplementary Figure S4).

**Figure 4:**
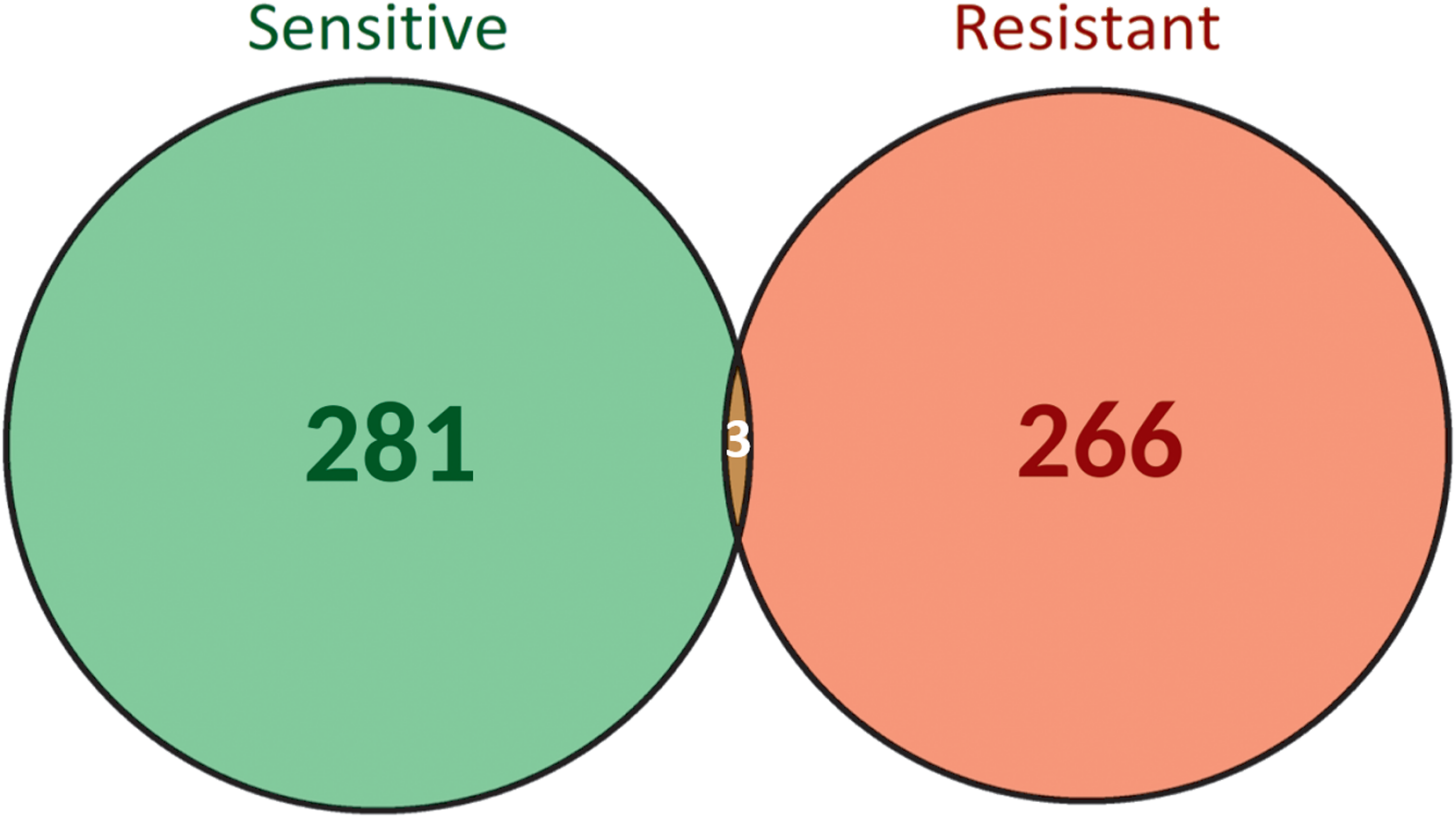
Biomarker-drug-tumour trios classification according to the type of response to treatment (sensitive or resistant).

Importantly, the same biomarker can be associated to resistance or sensitivity depending on the context of treatment and cancer type, highlighting the importance of considering the contextual information when assessing the effect of the biomarker. If we focus on the biomarker-drug-tumour associations, a clear separation between resistant (281) and sensitive (266) biomarkers is observed (Figure 4). Only three biomarker-drug-tumour combinations had both types of responses: the triplets ‘BRAF V600E-panitumumab, trametinib-colorectal cancer’, ‘EGFR R451C-cetuximab-colorectal cancer’ and ‘KRAS G13D-cetuximab-colorectal cancer’. These can be explained by differences in annotation between the source databases and by distinct evidence levels, respectively. For instance, in the ‘EGFR R451C-cetuximab-colorectal cancer’ example, the biomarker is associated to sensitivity to treatment at preclinical level but to resistance to treatment at clinical level. Therefore, it is important to take into account all the evidence reporting a biomarker when assessing its utility and potential application. This information is provided by ResMarkerDB.

**Figure 5:**
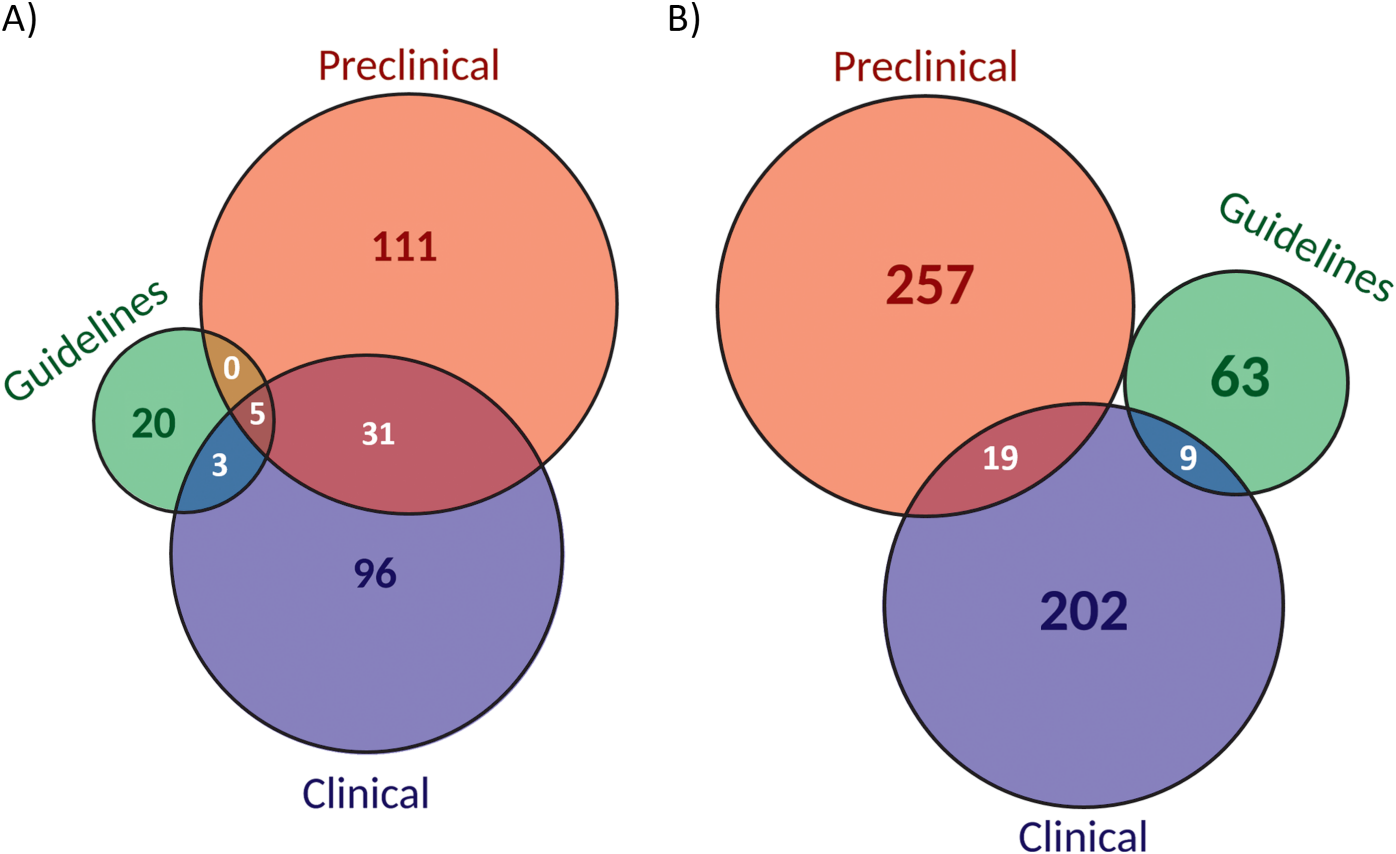
Classification of biomarkers (**A**) and biomarker-drug-tumour combinations (**B**) according to the evidence level (preclinical, clinical or guidelines).

The evidence level analysis (Figure 5A) showed that more than 50% of biomarkers were reported at clinical levels while 55% at preclinical levels. In contrast, only 11% were considered in guidelines. Moreover, 14% of the biomarkers were shared between clinical and preclinical levels and only 2% at the three levels. When considering biomarker-drug-tumour trios per evidence level, the percentage of combinations per evidence level was reduced to 42% at clinical levels, to 50% at preclinical levels and 13% in guidelines (Figure 5B). Interestingly, the overlap between the three levels completely disappears.

### Web interface

**Figure 6:**
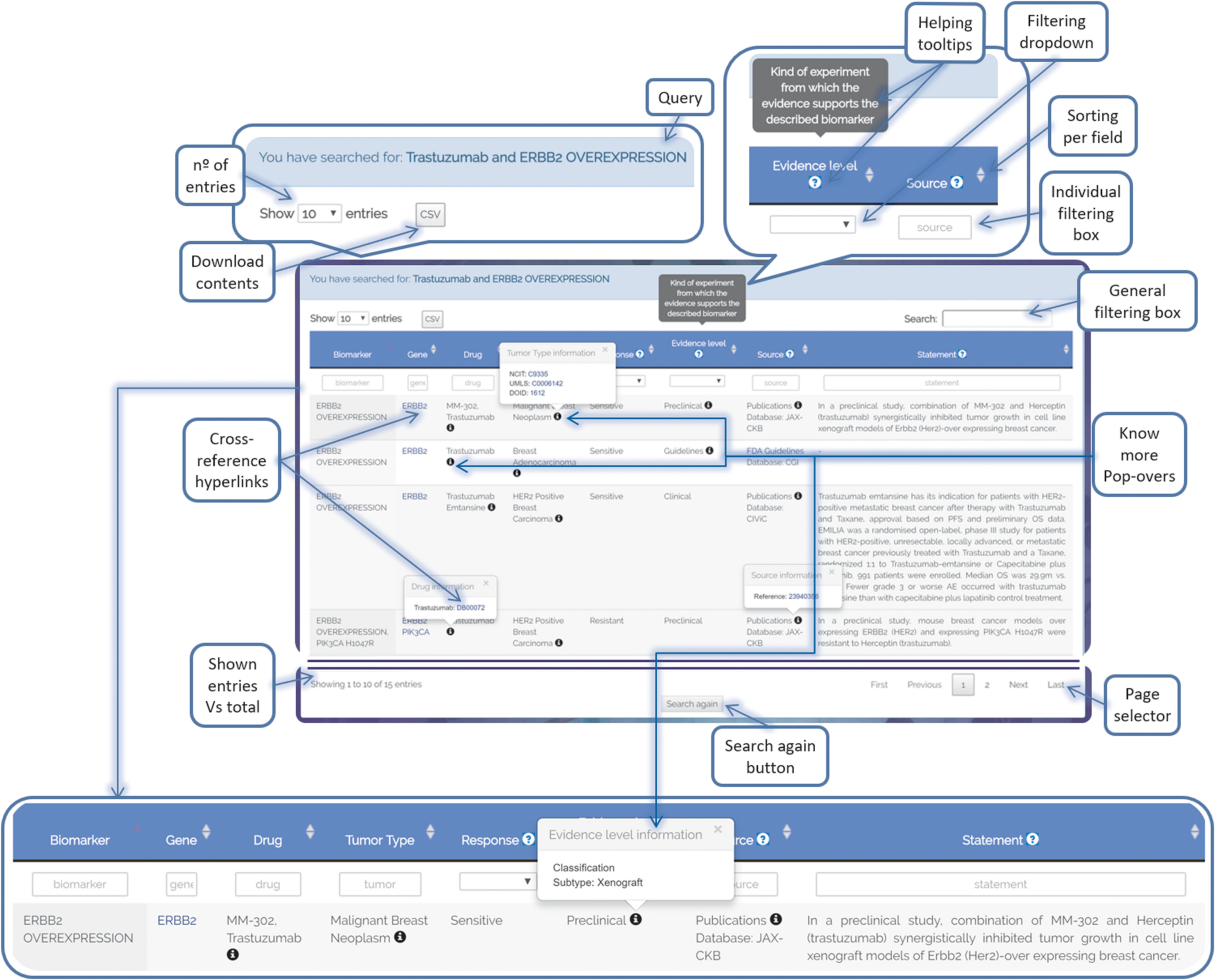
ResMarkerDB output page example illustrating the different functionalities that have been implemented to support user-friendly exploration of the data (e.g. cross-reference hyperlinks, know-more popovers, filters, download button, etc.).

ResMarkerDB search interface allows queries by biomarker, tumour type and/or drug by means of a text area and a dropdown menu. In addition, a browser interface is available for the visualization of all database contents. The results are output in tabular format, providing information on biomarker, gene, drug, tumour, response, level of evidence, source and statement (Figure 6). Each row represents a different combination of these data. Filtering options are available for each field and cross-reference links to Pubmed, DrugBank and NCIT are provided among others. All data are available for download, with the exception of JAX-CKB data due to license restrictions.

In addition, user guidelines (http://resmarkerdb.org/help/) are available with detailed information on the data provided, including the homogenization and standardization process, as well as database statistics.

### Case study

**Figure 7:**
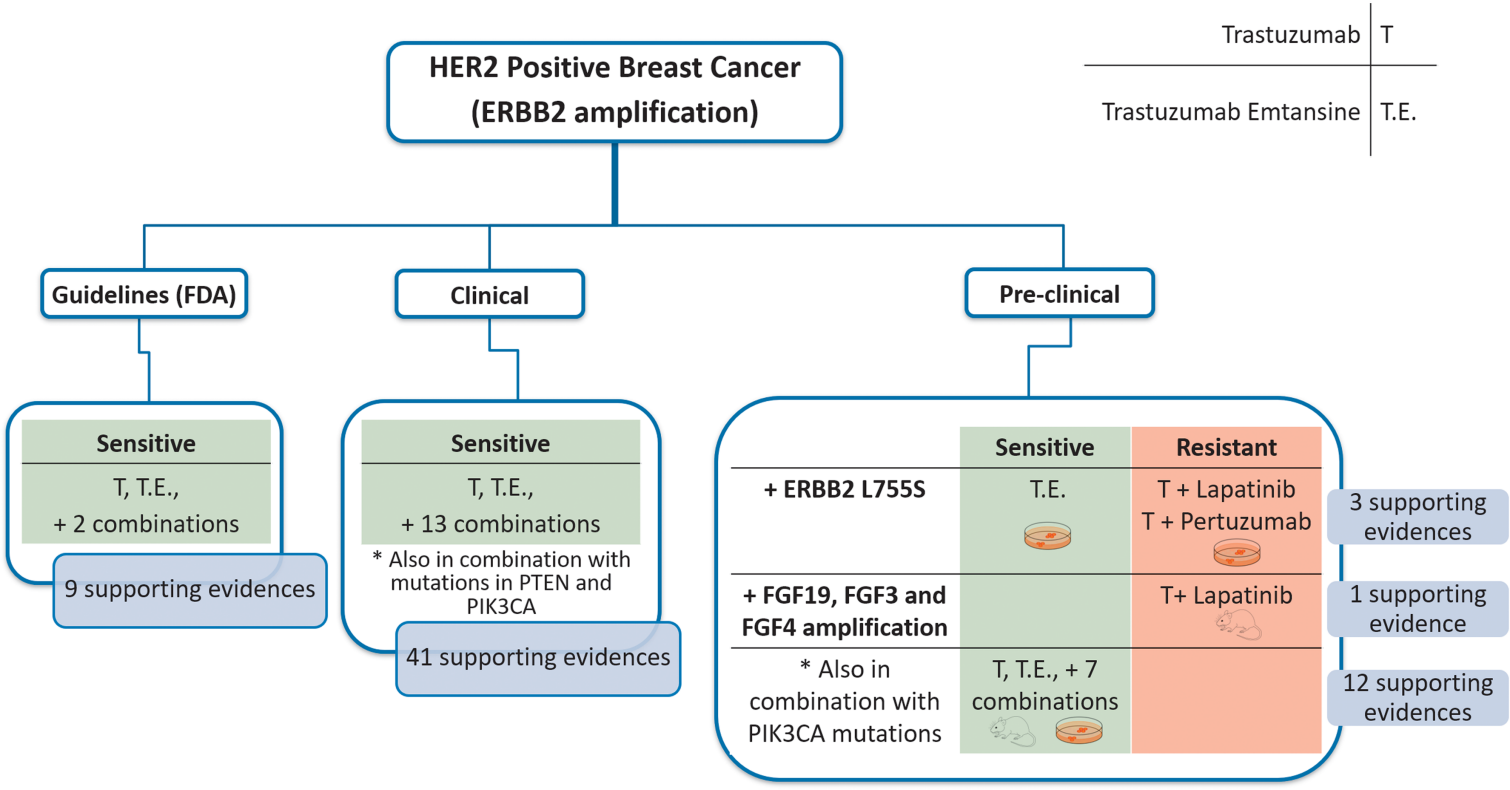
Schema of a case study (HER2 positive breast cancer) and the classifications made according to the evidence level (guidelines, clinical or preclinical), response (sensitive or resistant), biomarkers and treatments. There are 9 and 41 supporting evidences at guideline and clinical level, respectively, all of them reporting sensibility. Moreover, there are 15 evidences at preclinical showing different responses depending on the treatment or the presence of additional alterations.

We showcase the usefulness of the tool with a case study. Firstly, we analysed the use of ERBB2 amplification as a biomarker of response to different drugs (trastuzumab, pertuzumab and trastuzumab emtansine) in HER2 positive breast cancer (Figure 7). Of note, all these mAbs target ERBB2. ResMarkerDB contains records with different levels of supporting evidence for the above-mentioned biomarker and treatments: 9 records from guidelines, 41 records from clinical studies and 16 records from preclinical studies.

Regarding the guidelines, ERBB2 amplification is described as a biomarker of treatment sensitivity to trastuzumab, trastuzumab emtansine and some combination treatments.

Considering the clinical studies, ERBB2 amplification was reported as a biomarker of treatment sensitivity with these mAbs, both alone or in combination with other antineoplastic or immunomodulating agents. In these studies, sensitivity is reported in the presence of ERBB2 amplification in combination with other alterations (PTEN or PIK3CA mutations). PIK3CA is an oncogene that acts downstream to the ERBB2 signalling pathway. On the contrary, PTEN inhibits ERBB2 signalling pathway and is frequently underexpressed or deleted, acting as a tumour suppressor. Thus, when a PIK3CA activating mutation or a PTEN inactivating mutation is in combination with ERBB2 amplification, a decreased progression-free survival is reported, and therefore, a combination treatment is suggested (30). Thus, in these cases, it is important to consider the statement supporting the association where these nuances can be explored.

Finally, at preclinical level, we find different responses depending on the biomarker but also on the particular treatment. If ERBB2 amplification is found in combination with amplification of growth factors FGF19, FGF3 and FGF4, there is evidence of resistance to trastuzumab and lapatinib treatment in xenografts. Lapatinib is a tyrosine kinase inhibitor and it has been reported that a higher availability of growth factor ligands may alter the cancer cells sensitivity to them (31). On the other hand, ERBB2 amplification alone confers sensitivity to trastuzumab in combination with other treatments in cell lines and xenografts. Interestingly, ERBB2 amplification in combination with activating mutations in PIK3CA is associated to sensitivity to trastuzumab emtansine without affecting its efficacy. Owing to its cytotoxic moiety, trastuzumab emtansine can bypass intracellular signalling pathways downstream ERBB2 and keep its functionality when some resistance mechanisms exist (32). In addition, sensitivity to trastuzumab emtansine and resistance to trastuzumab + lapatinib and trastuzumab + pertuzumab is reported for ERBB2 amplification in combination with ERBB2 L755S in specific cell lines. However, the role of this mutation in driving the resistance to treatment is not completely understood (33).

In summary, for HER2 positive breast cancer, ERBB2 amplification alone is reported as a sensitivity biomarker for the treatment with trastuzumab, trastuzumab emtansine and other combination treatments in clinical guidelines, while in clinical and preclinical studies, combination of ERBB2 amplification with other biomarkers are being investigated in different experimental settings and with different drug combinations.

## Limitations

Despite the value of ResMarkerDB in offering data on biomarkers of response to mAbs in breast and colorectal cancer, it is worth considering some limitations of the resource. First, the text mining process applied is focused on abstracts and on single nucleotide polymorphism and ncRNAs associated to sensitivity or resistance to mAbs in breast or colorectal cancer. Therefore, other kind of biomarkers of response or those not stated in the abstract but in other sections of a publication could not be captured. Future efforts on updating the database will likely extend the scope of biomarkers recovered by text mining.

The second limitation concerns the narrow coverage of cancer types and therapies of the current version of ResMarkerDB. The platform was developed in response to a request from a clinical oncology unit specialized in breast and colorectal cancer. Despite this focused field of application, the approach developed to implement ResMarkerDB has proven to be useful for a wider application in the context of precision oncology. More specifically, it was used to support the retrieval of articles reporting tailored treatments for cancer patients with specific genomic alterations by a text mining process, in the context of the ‘2018 TREC Precision Medicine/Clinical Decision Support Track’ (http://www.trec-cds.org/2018.html). This dataset is available at the ResMarkerDB web page (http://resmarkerdb.org/help/#challenge_data).

The third limitation is the incomplete characterization of the original data regarding some clinical features of the tumour or the treatment. In this sense, neither the source databases nor ResMarkerDB are able to collect more specifications on the tumour type (e.g. tumour site location in colorectal cancer) or treatment (e.g. clinical setting of the prescription, for instance adjuvant or primary treatment). In addition, currently there is no available information in the source databases about biomarkers of response for all the clinically relevant cancer subtypes. This could be a consequence of the lack of characterization of biomarkers for particular disease subtypes and treatments or due to terminology or annotation issues.

Finally, ResMarkerDB data relies on the integrity of its source databases. These databases have been chosen because they are well-established resources that have been regularly updated and have been available during the past years. However, these data from third parties are complemented and augmented with our own efforts in text mining and curation of data from the literature.

## Discussion and conclusions

ResMarkerDB aims to enhance translational research efforts in identifying existing and new actionable biomarkers of drug response in cancer and benefit from them. Its usefulness lies in the possibility to prioritize biomarker data in the context of response to therapy in a specific cancer type according to the evidence supporting the association and its potential clinical actionability. To the best of our knowledge, there is no other repository dedicated only to the study of biomarkers of drug response in these cancer types.

The relatively small overlap between databases stems from differences in the terminology used to describe the cancer types and treatments and in the annotation of biomarkers. Altogether, it shows the need of a centralized repository that gathers biomarkers of response to mAbs for breast and colorectal cancer. ResCur data, obtained by text mining followed by expert curation, revealed 67 new biomarkers, 12 new treatments and 89 new biomarker-drug-tumour combinations not previously reported in any of the source databases. This brings to light the fact that there are still data present in the literature that need to be properly annotated and considered to promote the discovery of new actionable biomarkers. Also, the data in ResCur have been annotated with higher granularity, providing more detailed information regarding the specific cancer type, which was not available in the other sources. Additionally, ResCur contains information about the exact alteration of ncRNA biomarkers that was not initially reported by the source databases. More concretely, changes in gene expression of miRNAs and long non-coding RNAs (lncRNAs) in relation to treatment response in breast and/or colorectal cancer support the role of ncRNAs as biomarkers of drug response in these cancer types.

ResMarkerDB usefulness also lies in the detailed provenance of the information provided to the user, which can be used to filter and prioritize biomarkers. While basic researchers may be interested on evidences from preclinical studies, clinical practitioners might give more value to evidences from clinical studies and guidelines. Of note, there are contradictory responses for the same biomarker-drug-tumour trio (Figure 4). This could be explained by different experimental methods leading to different conclusions. Fifty percent of biomarker-drug-tumour combinations are reported by preclinical studies while 42% are evidences from clinical studies (Figure 5). In addition, we have shown by means of a case study the importance of looking at the provided context for treatment response, which is treatment, cancer type and level of evidence.

In summary, ResMarkerDB is a publicly available resource that aims to facilitate the exploration of current knowledge of predictive biomarkers of response to mAbs in breast and colorectal cancer.

## Supplementary Material

ResMarkerDB_suppl_rev1_baz060

## Notes

**Database URL**: http://www.resmarkerdb.org
