# Supplementary material for: ResMarkerDB: a database of biomarkers of response to antibody therapy in breast and colorectal cancer
Source: Database (Oxford). 2019 Jun 4;2019:baz060. doi: 10.1093/database/baz060 (PMC6551372; doi:10.1093/database/baz060)
Supplement: ResMarkerDB_suppl_rev1_baz060 [file resmarkerdb_suppl_rev1_baz060.docx]

ResMarkerDB: a database of biomarkers of response to antibody therapy in breast and colorectal cancer

# Supplementary material contents

**Supplementary Figure S1.** Biomarkers, Drugs and Biomarker-Drug-Tumor trios classification per source.

**Supplementary Figure S2.** Prevalence in TCGA cohorts of genes in ResMarkerDB.

**Supplementary Figure S3.** Biomarkers classification per cancer type and per source.

**Supplementary Figure S4.** ncRNAs association to breast and colorectal cancer.

**Supplementary Figure S5.** Biomarkers classification per evidence level.

**Supplementary Table S1.** Databases’ feature comparison.

**Supplementary Table S2.** Classification of drugs according to the Anatomical Therapeutic Chemical (ATC) classification system, group “L antineoplastic and immunomodulating agents”.

**Supplementary Table S3.** Enrichment analysis of biological processes in the Gene Ontology.

**Supplementary Figure S1.** Biomarkers, Drugs and Biomarker-Drug-Tumor trios classification per source.

| 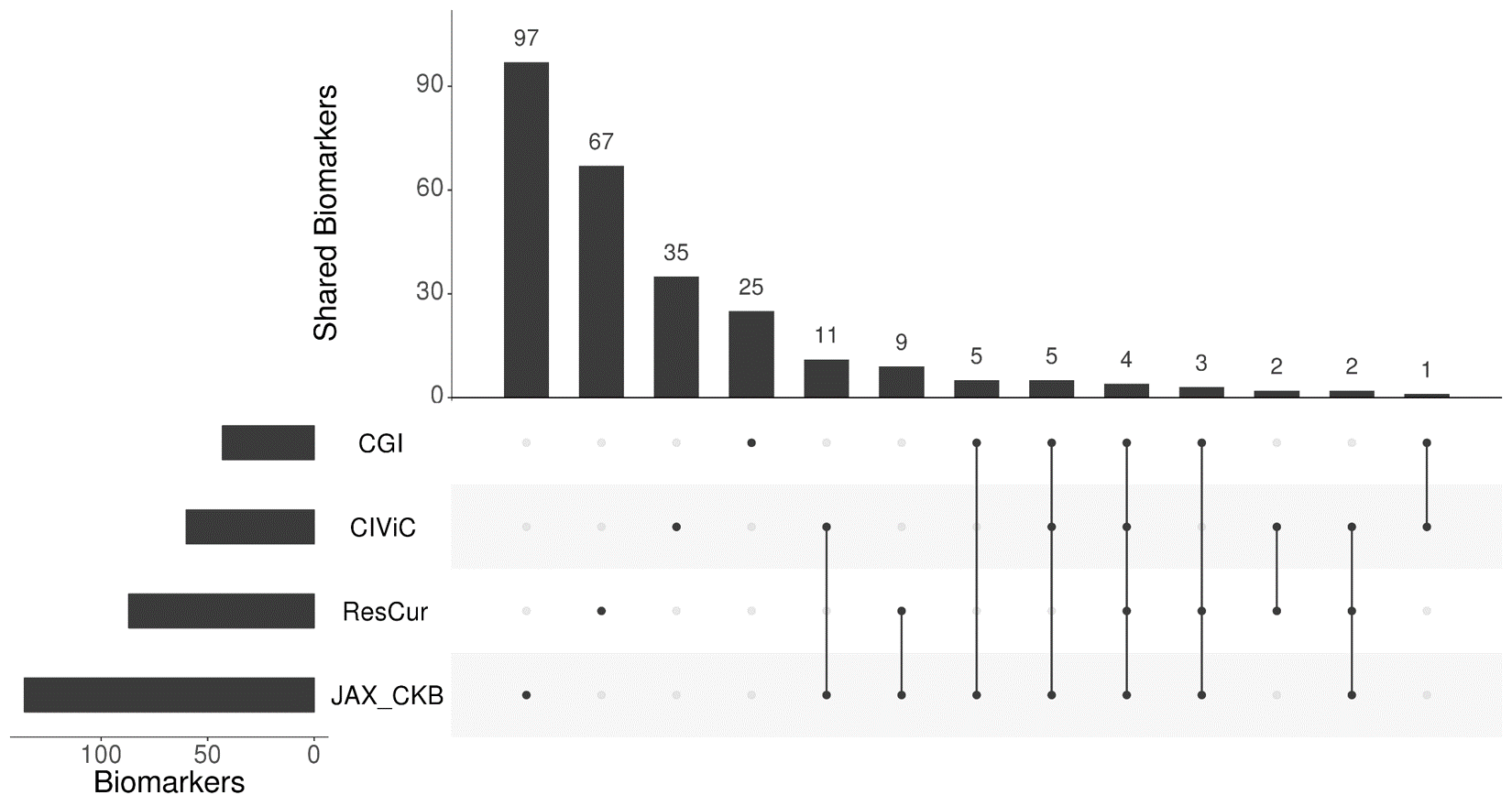A) |
| --- |
| 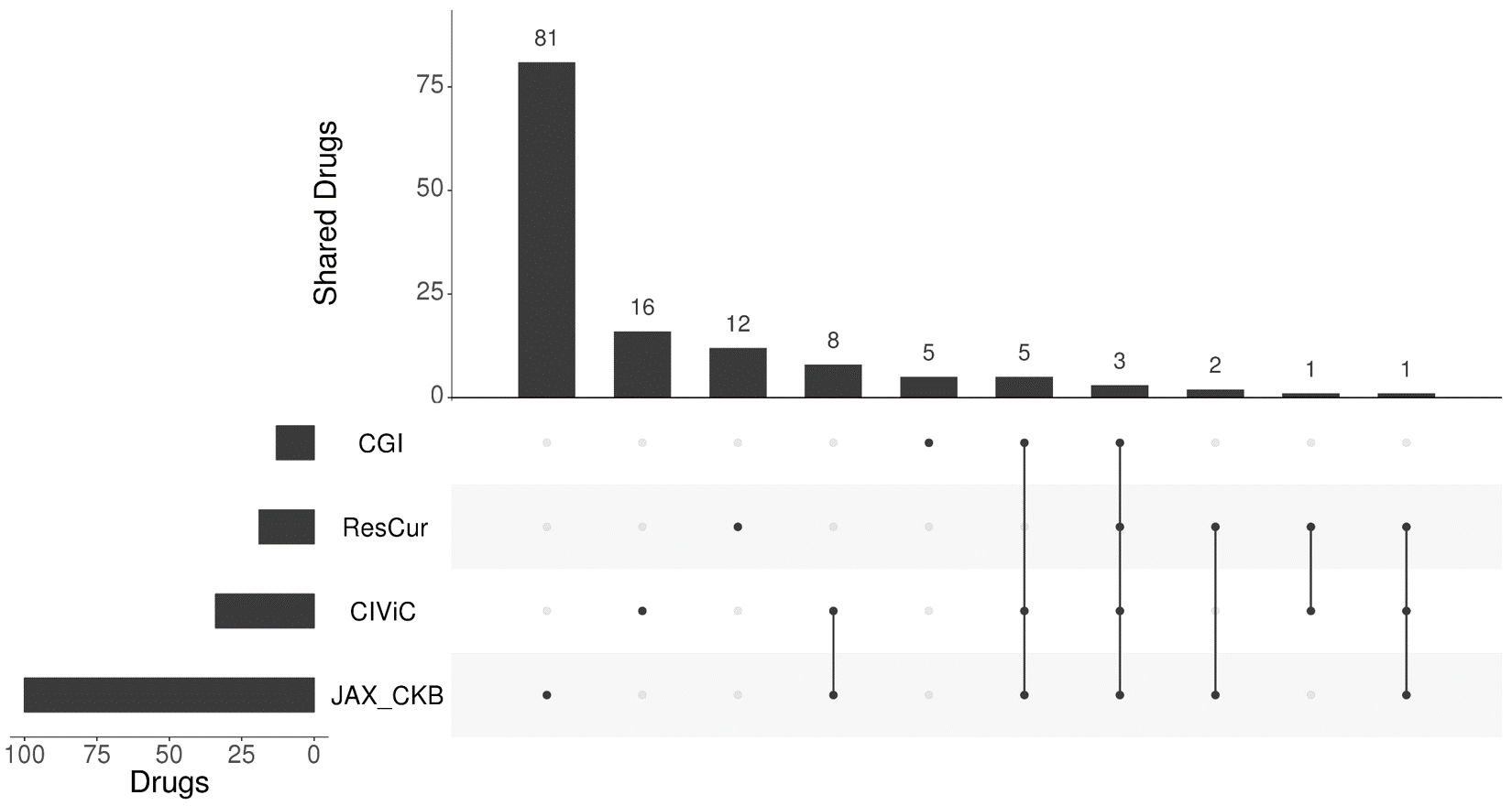B) |
| 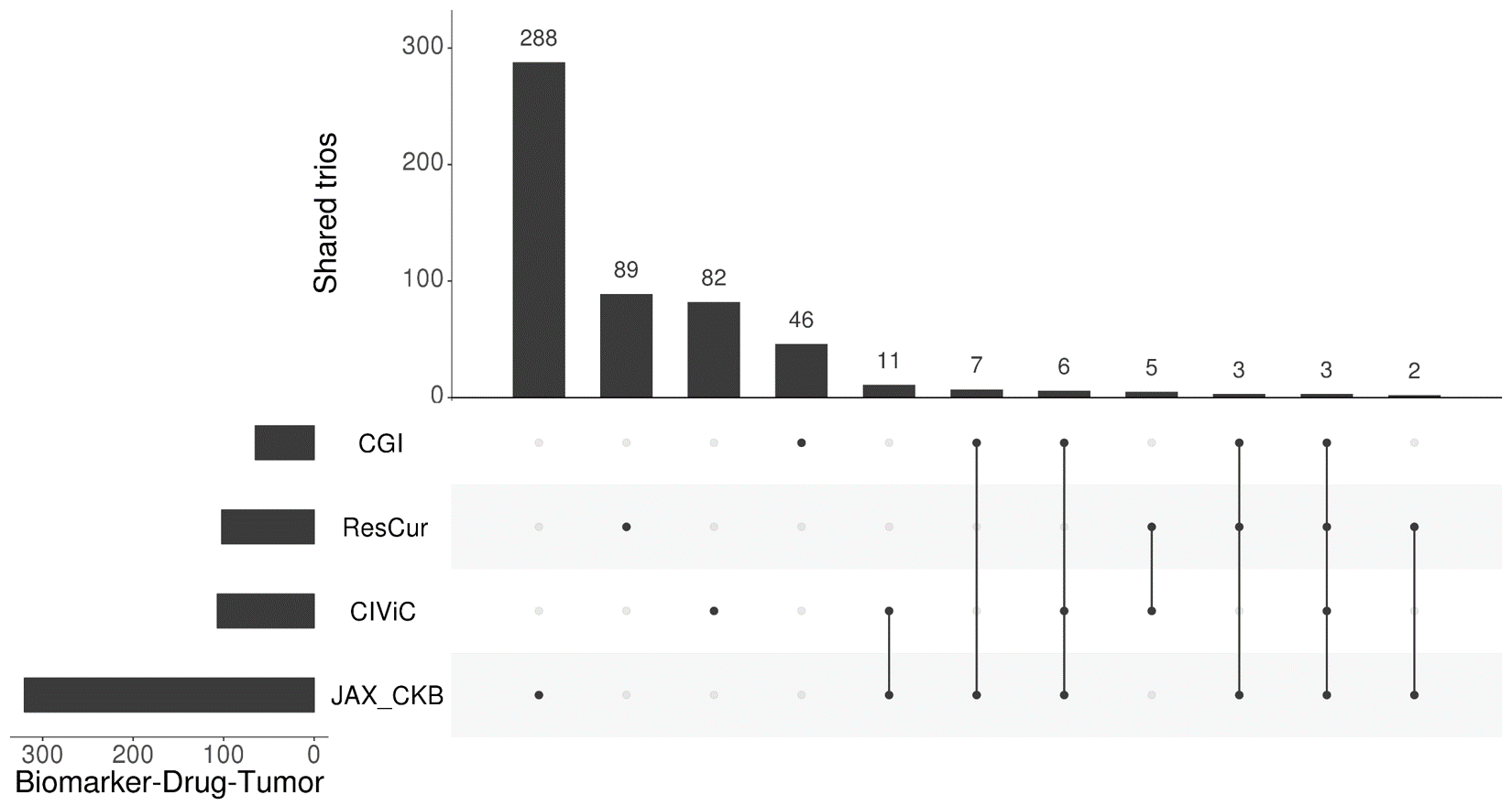C) |

Classification according to the reporting source: CGI, CIViC, JAX-CKB or ResCur**:** A) Biomarkers, B) Drugs and C) Biomarker-drug-tumor trios. CGI: Cancer Genome Interpreter, CIViC: Clinical Interpretations of Variants in Cancer, JAX-CKB: JAX-Clinical Knowledgebase.

**Supplementary Figure S2.** Prevalence in TCGA cohorts of genes in ResMarkerDB.

| 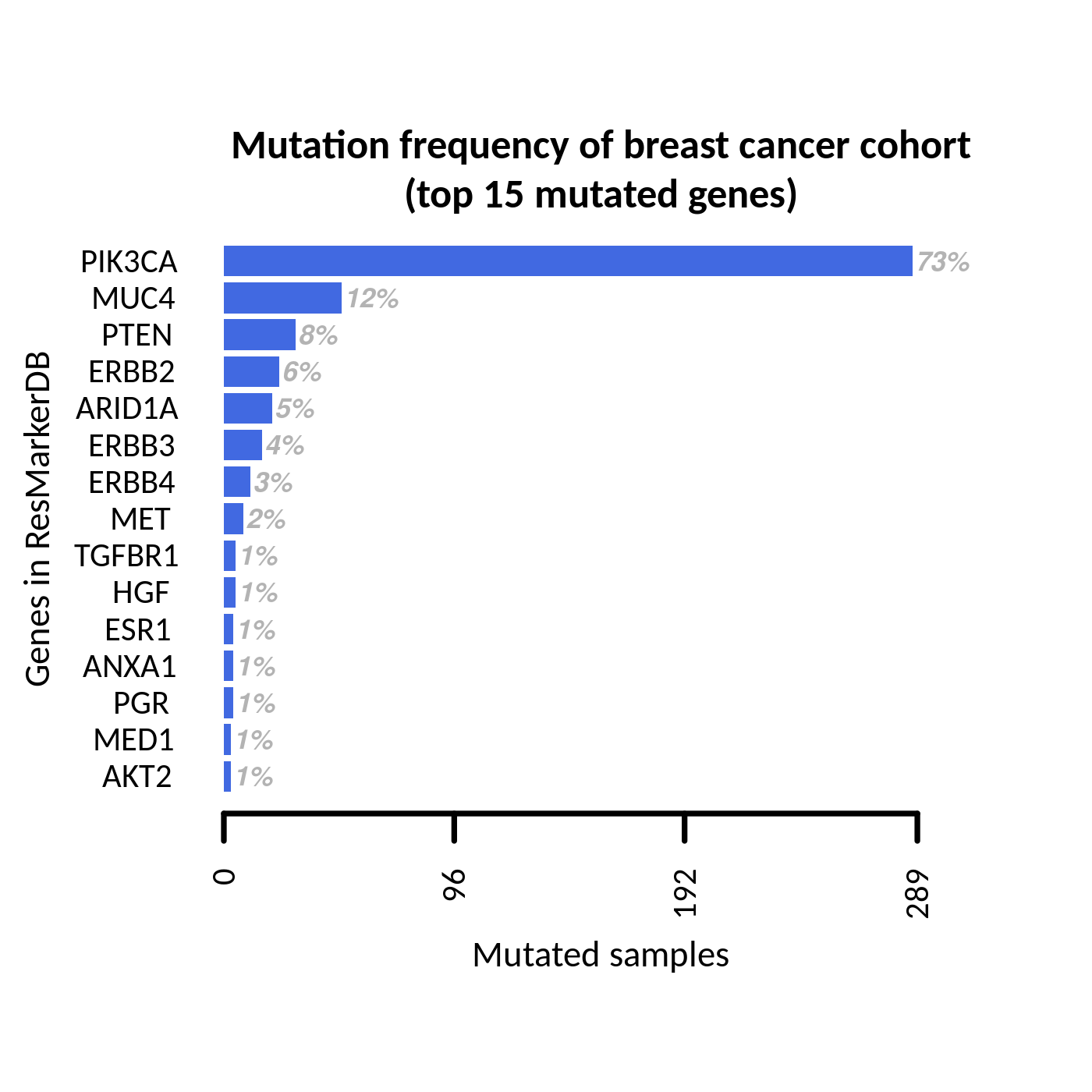A) |
| --- |
| 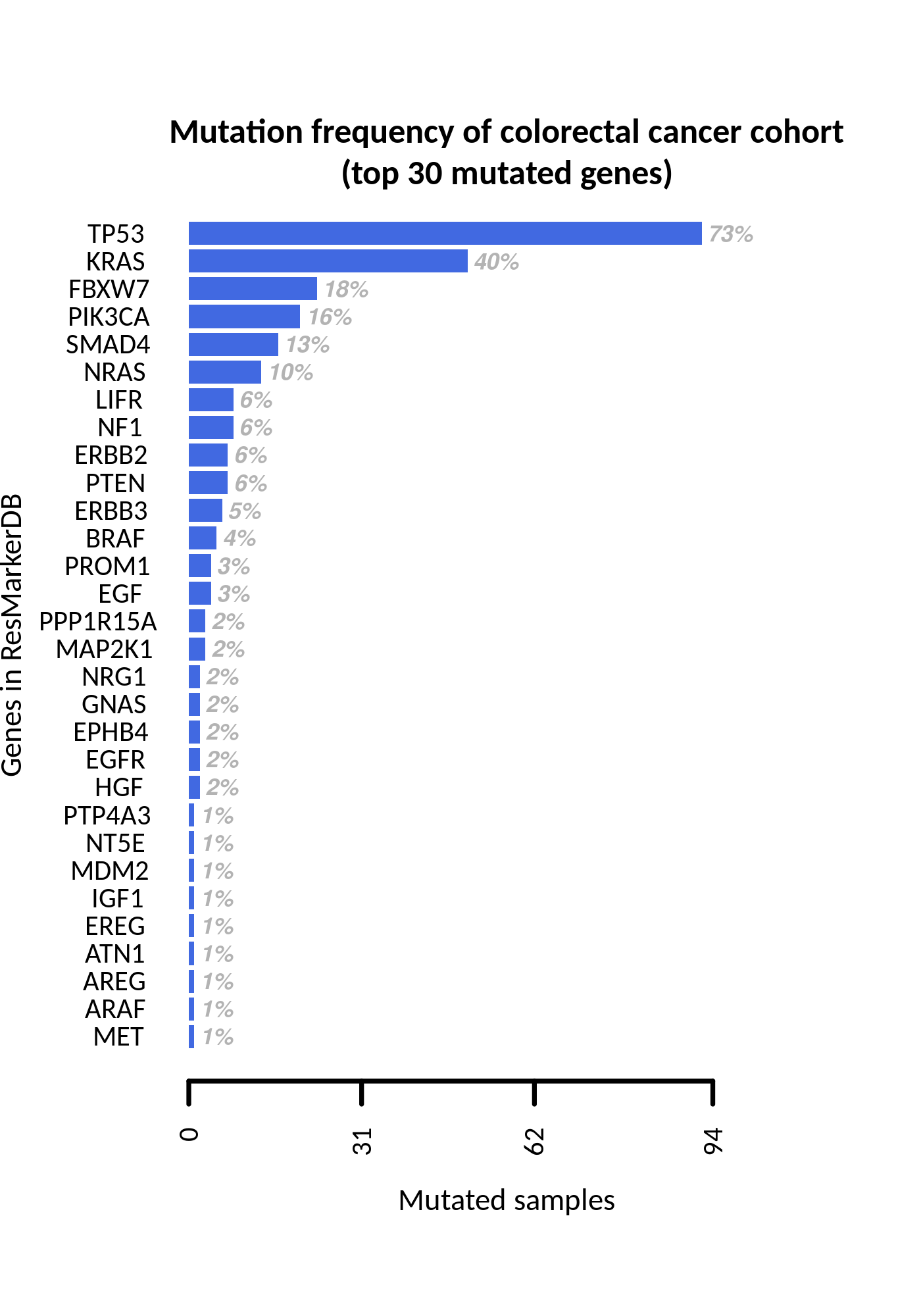B) |

Prevalence in the TCGA cohorts (A- breast cancer cohort and B- colorectal adenocarcinoma cohort) of the genes contained in ResMarkerDB. The bars represent the number of samples (patients) harboring alterations in ResMarkerDB genes in the TCGA cohorts (A: breast cancer cohort, B: colorectal adenocarcinoma cohort). Only the top 15 and top 30 mutated genes are shown. The percentage of samples (patients) for each gene is shown at the right side of each bar.

**Supplementary Figure S3.** Biomarkers classification per cancer type and per source.

| A)  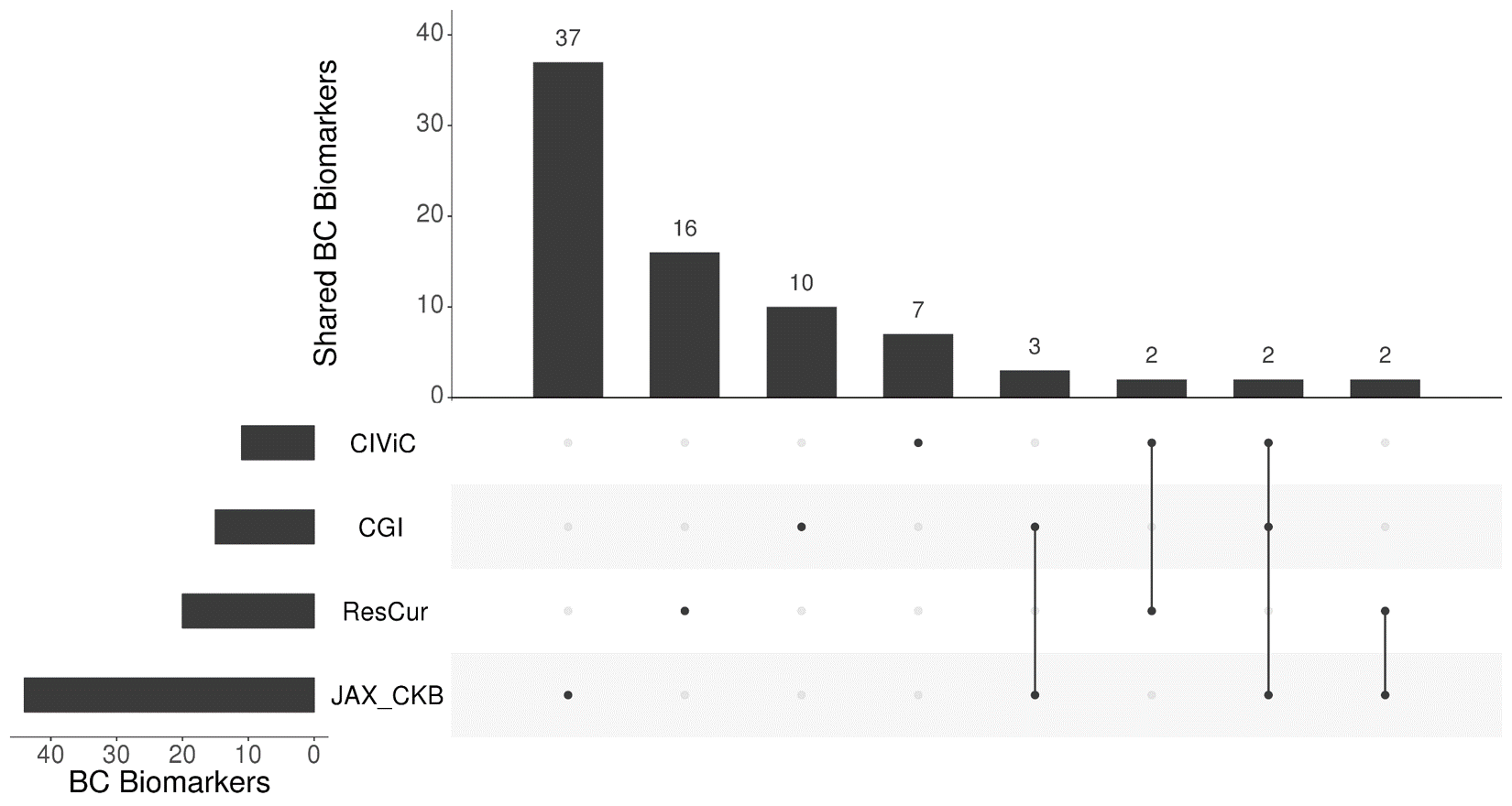 |
| --- |
| B)  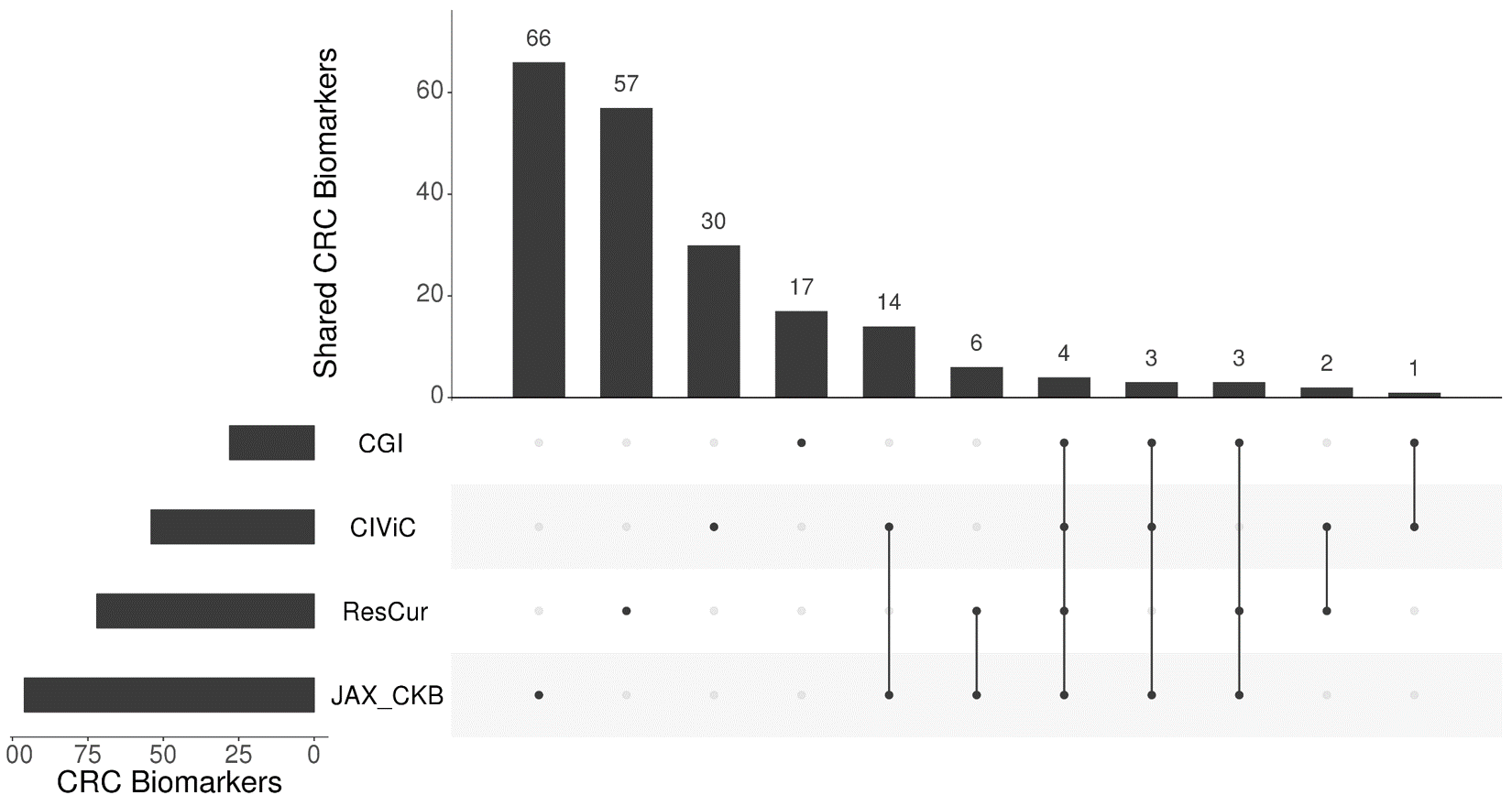 |

Distribution of biomarkers per tumor type (A- breast cancer or BC and B- colorectal cancer or CRC) according to the source reporting the association: CGI, CIViC, JAX-CKB or ResCur. CGI: Cancer Genome Interpreter, CIViC: Clinical Interpretations of Variants in Cancer, JAX-CKB: JAX-Clinical Knowledgebase.

|  |
| --- |

**Supplementary Figure S4.** ncRNAs association to breast (A) and colorectal cancer (B).

A)

| **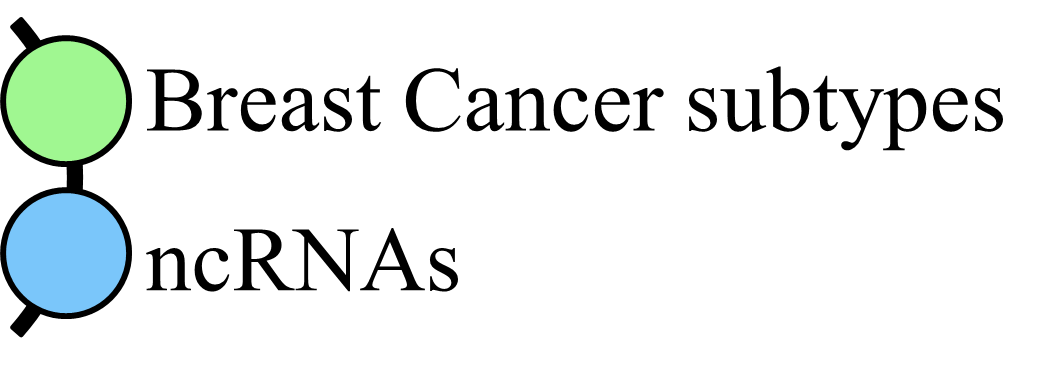**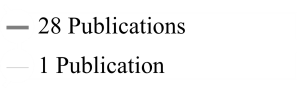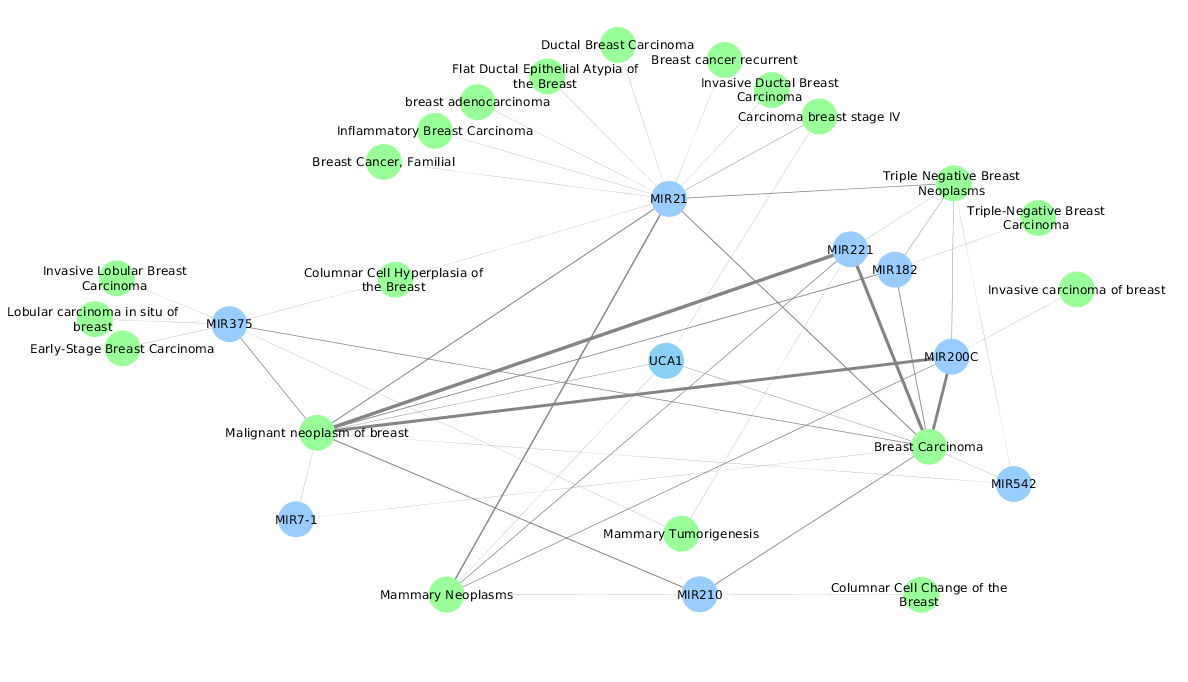 | |
| --- | --- |
| 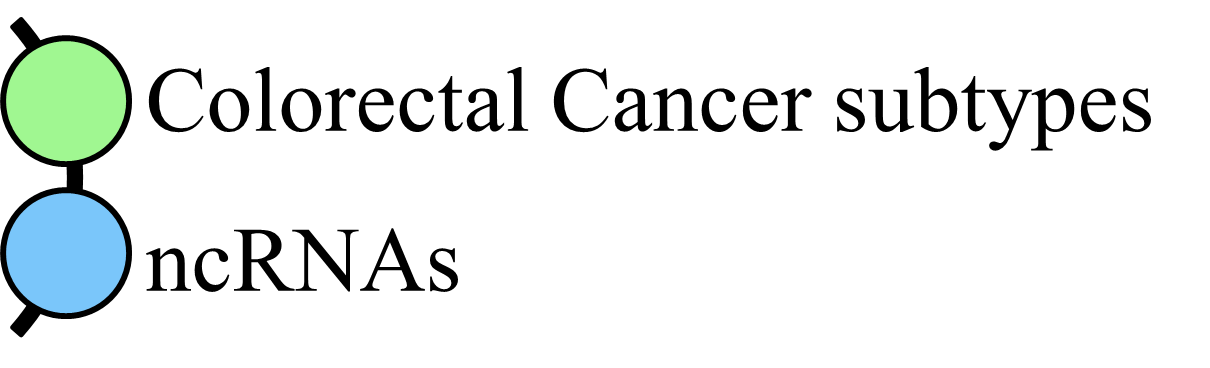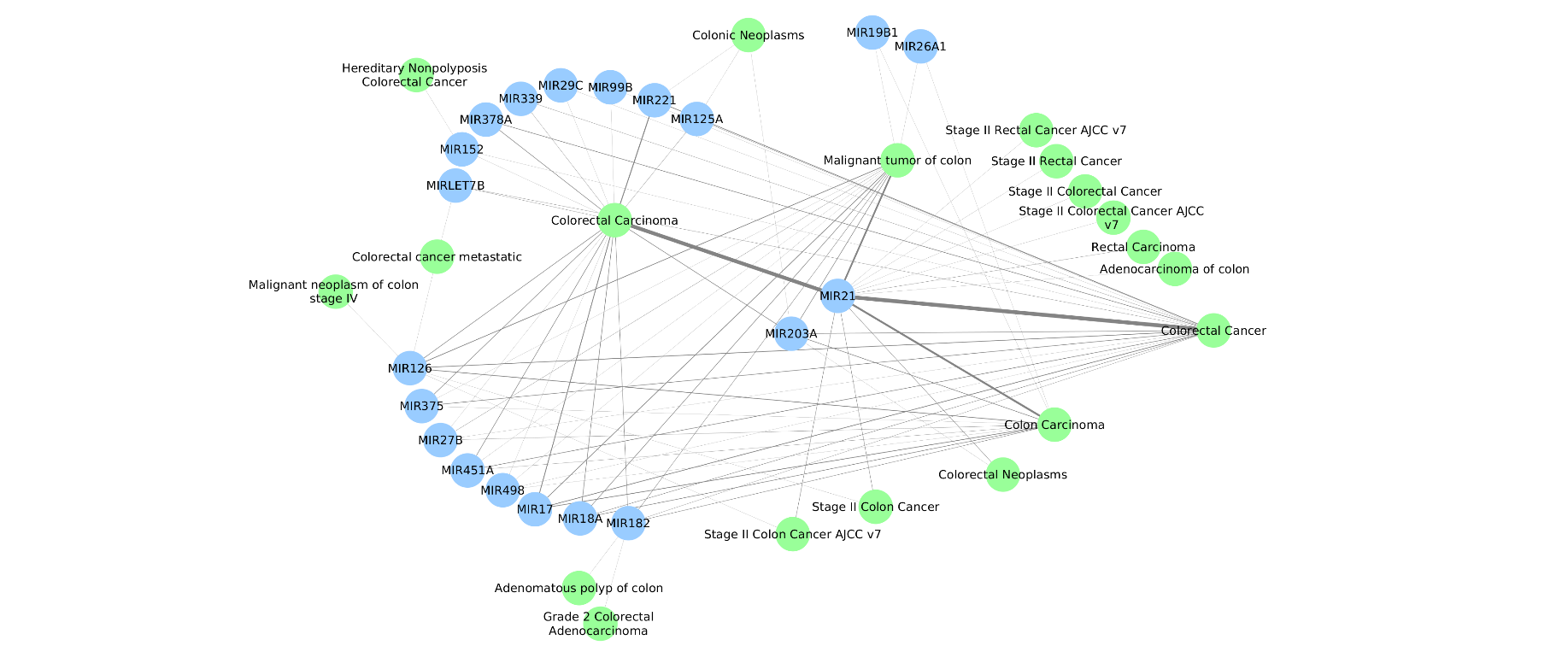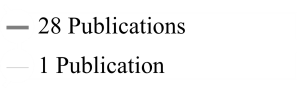B) |  |

Gene-disease association network showing ncRNAs (blue circles) contained in ResMarkerDB associated to each cancer subtypes (A- breast cancer and B- colorectal cancer) (green circles). The edges thickness is proportional to the number of publications reporting the association, ranging from 1 to 28.

**Supplementary Table S1.** Databases’ feature comparison

Feature comparison of the source databases and ResMarkerDB. *Number of entries taking into account those that match our scope, meaning FDA-approved therapeutic monoclonal antibodies for breast and colorectal cancer. We consider an entry the unique combinations of biomarker, drug, tumor type, response, evidence level, source and statement.

| Feature | CIViC | CGI | JAX-CKB | ResMarkerDB |
| --- | --- | --- | --- | --- |
| Approximate size* | 140 entries | 67 entries | 336 entries | 656 entries |
| Content description | Structured vocabulary | Structured vocabulary | Structured vocabulary | Structured controlled, hierarchical and standardized vocabulary |
| Biomarkers type | Coding genes | Coding genes | Coding genes | Coding genes and non-coding RNAs |
| Referenced to original publication | Yes | Yes | Yes | Yes |
| Active curation | Yes | Yes | Yes | Yes |
| Accessibility | Website and API | Website and REST API | Website | Website |
| Downloadable contents | Yes | Yes | No | Yes (except for JAX-CKB data) |
| Website | https://www.cancergenomeinterpreter.org/biomarkers | https://civicdb .org/releases | https://ckb.jax .org | http://resmarkerdb .org/browse/ |

**Supplementary Table S2**: Classification of drugs according to the Anatomical Therapeutic Chemical (ATC) classification system, group “L antineoplastic and immunomodulating agents”.

| **ANTINEOPLASTIC AND IMMUNOMODULATING AGENTS** | |
| --- | --- |
| ANTINEOPLASTIC AGENTS | |
| Alkylating agents | 1 |
| Antimetabolites | 6 |
| Plant alkaloids and other natural products | 3 |
| Cytotoxic antibiotics and related substances | 2 |
| Other antineoplastic agents | 54 |
| Monoclonal antibodies | 9 |
| Protein kinase inhibitors | 38 |
| Other antineoplastic agents | 6 |
| Combinations of antineoplastic agents | 1 |
| Total | 66 |
| ENDOCRINE THERAPY | |
| Hormone antagonists and related agents | 4 |
| Total | 4 |
| IMMUNOSUPPRESSANTS | |
| Immunosuppressants | 1 |
| Total | 1 |

**Supplementary Table S3.**  Enrichment analysis of biological processes in the Gene Ontology.

Statistical overrepresentation test of the database genes according to GO-Slim Biological Process. For each category, the number of *Homo sapiens* genes for that category (total annotated genes), the observed ones in our gene set, the expected ones, the enrichment direction (over or under), the computed fold enrichment, p-value and Benjamini-Hochberg False Discovery Rate (FDR) are referred.

| PANTHER GO-Slim Biological Process | Total Genes Annotated | Observed | Expected | Fold  Enrichment | p-value | FDR |
| --- | --- | --- | --- | --- | --- | --- |
| MAPK cascade (GO:0000165) | 340 | 13 | 0,92 | 14,11 | 8,15E-12 | 9,94E-10 |
| negative regulation of apoptotic process (GO:0043066) | 99 | 6 | 0,27 | 22,37 | 3,88E-07 | 1,58E-05 |
| transmembrane receptor protein tyrosine kinase signaling pathway(GO:0007169) | 151 | 6 | 0,41 | 14,67 | 4,09E-06 | 9,98E-05 |
| regulation of catalytic activity (GO:0050790) | 359 | 8 | 0,97 | 8,23 | 6,10E-06 | 1,35E-04 |
| regulation of phosphate metabolic process (GO:0019220) | 537 | 9 | 1,45 | 6,19 | 1,45E-05 | 2,95E-04 |
| I-kappaB kinase/NF-kappaB cascade (GO:0007249) | 45 | 3 | 0,12 | 24,61 | 2,97E-04 | 3,62E-03 |
| cell differentiation (GO:0030154) | 548 | 7 | 1,48 | 4,72 | 7,10E-04 | 8,25E-03 |
| angiogenesis (GO:0001525) | 18 | 2 | 0,05 | 41,02 | 1,32E-03 | 1,40E-02 |
| receptor-mediated endocytosis (GO:0006898) | 104 | 3 | 0,28 | 10,65 | 3,04E-03 | 2,97E-02 |
